# Histiocytoid variant of invasive lobular breast carcinoma. A case report and literature review

**DOI:** 10.1016/j.amsu.2021.103091

**Published:** 2021-11-18

**Authors:** Fozan A. Aldulaijan, Abdullah G. Alsahwan, Maryam Hussain A. Alsulaiman, Miral Mohamed Mashhour, Ahmad Alwabari

**Affiliations:** a Department of Surgery, Breast and Endocrine Surgery Section, King Fahad Specialist Hospital‐Dammam, Dammam, Saudi Arabia; b Department of Adult Oncology, King Fahad Specialist Hospital, Dammam, Saudi Arabia; c Department of Pathology and Laboratory Medicine, King Fahad Specialist Hospital, Dammam, Saudi Arabia

**Keywords:** Histiocytoid breast carcinoma, Invasive breast carcinoma, Invasive lobular carcinom

## Abstract

**Introduction:**

Histiocytoid breast carcinoma (HBC) is a variant of invasive lobular carcinoma. The occurrence of HBC is rare and the natural history and clinical course of HBC is still not well known due to limited numbers of reported cases. In reality, many tumors have been misdiagnosed and reported as benign lesions.

**Case presentation:**

A 66-year-old- postmenopausal women, who has previous personal history of right breast invasive ductal carcinoma, for which she underwent right breast wide local excision with negative sentinel lymph node biopsy and received adjuvant radiotherapy and hormonal therapy. Two years later, a new left breast suspicious lesion was detected by Imaging. Breast Ultrasound showed left breast hypo-echoic area at 12-1 o'clock with irregular spiculated lesion 3 cm away from the nipple with posterior acoustic shadowing measuring 1 × 0.7 × 0.7 cm and mild tissue distortion with thicken cortical left Axillary lymph node. Mammography of both breasts confirmed the left breast lesion at 12o'clock with necrosis and irregular margins measuring 1.1 × 1.0 cm. MRI breasts showed, left breast heterogeneously enhancing mass at 12 o'clock with no other suspicious mass in the left or right breast. Ultrasound guided left breast biopsy of the suspicious lesion seen at 12-1 o'clock which confirmed the diagnosis of invasive lobular carcinoma, histiocytoid variant She underwent wire guided left breast wide local excision with left sentinel lymph node and axillary clearance. Final histopathology showed invasive lobular carcinoma, histiocytoid variant.

**Clinical discussion:**

The recognition of histiocytoid breast carcinoma is often a challenge, particularly when histiocytoid tumor cells occur in a metastatic site before the primary diagnosis of breast cancer. An awareness of histological features are needed to make the accurate diagnosis.

**Conclusion:**

Findings that support the correct diagnosis include identifying tumor cells with more cytological atypia, the presence of cytoplasmic vacuoles and secretions. Moreover, coexistence with invasive lobular carcinoma and/or lobular neoplasia and the use of immunohistochemistry to confirm their epithelial nature. clinico-radiological correlation is essential, as any discordance should trigger further diagnostic determination.

## Highlights

•Histiocytoid breast carcinoma (HBC) is a rare variant of invasive lobular carcinoma.
•An awareness of histological features are needed to make the accurate diagnosis.
•Metastases to skin can be missed as benign sinus histiocytes and xanthomatous dermal lesions.
•Close clinic-radiological correlation is essential, as any discordance should trigger further diagnostic determination.

## 1 Introduction

Histiocytoid breast carcinoma (HBC) is a rare variant of invasive lobular carcinoma. The natural history and clinical course of HBC is still not well known due to limited numbers of reported cases [1]. It can be misdiagnosed due to overlapping histologic features with benign and other malignant lesions [2]. In 1973, the first reported case of HBC was described by Hood et al. [3]. After reviewing the English literature, 40 cases with the diagnoses of HBC were reported. Herein, we report and present a case which was diagnosed with this rare type of breast cancer.

The work has been reported in line with the SCARE 2020 criteria [13].

## 2 Case presentation

**Fig. 1:**
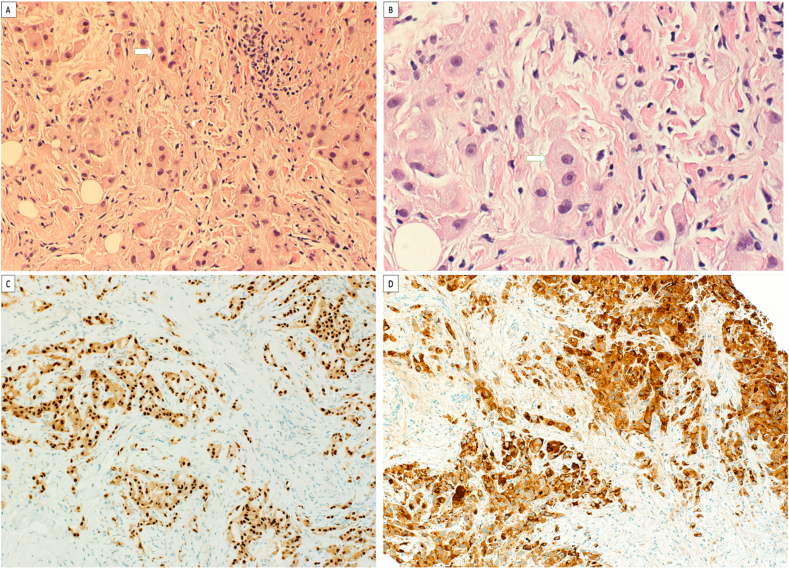
Pathological findings. **A.** Biopsy revealed Invasive histiocytoid carcinoma consisting of tumor cells with abundant granular cytoplasm (H/E)10X (arrows shown). **B.** Some of the nuclei are irregular (H/E)20X.**C.** Androgen receptor positive immunohistochemical stain. **D.** GCDFP-15 positive immunohistochemical stain.

A 66-year-old- postmenopausal women, who has no history of oral contraceptive use and no family history of breast cancer. Past history of right breast invasive ductal carcinoma, Grade II (pT1cN0M0) ER/PR positive, Her-neu2 Negative, for which she underwent right breast wide local excision with negative sentinel lymph node biopsy (five lymph nodes were identified all negative for malignancy) and received adjuvant radiotherapy and hormonal therapy. Two years following the procedure, a new left breast suspicious lesion was detected by Imaging. Clinically she had a right breast scar from previous surgery, but no palpable masses were felt in both breasts and axilla. Bilateral breast Ultrasound showed left breast hypo-echoic area at 12-1 o'clock with irregular spiculated lesion 3 cm away from the nipple with posterior acoustic shadowing measuring 1 × 0.7 × 0.7 cm and mild tissue distortion with thicken cortical left Axillary lymph node. The right breast showed tissue distortion at the previous surgical scar with no detectable cystic or solid lesion. Mammography of both breasts confirmed the left breast lesion at 12 o'clock with necrosis and irregular margins measuring 1.1 × 1.0 cm and no suspicious enhancing lesion on the right breast. MRI breasts showed, left breast heterogeneously enhancing mass at 12 o'clock with no other suspicious mass in the left or right breast. Staging CT scan of the chest, abdomen, and pelvis showed no evidence of metastatic disease. Bone scan showed no evidence of bone metastasis. Ultrasound guided left breast biopsy of the suspicious lesion seen at 12-1 o'clock which confirmed the diagnosis of invasive lobular carcinoma, histiocytoid variant with no ductal carcinoma in situ, no lympho-vascular and perineural invasion. Immunoprofile as follows, ER: negative, PR: negative, HER2-NEU: equivocal (score +2) FISH negative, E-cadherin: negative (granular), Ki-67: 10%. Other left breast biopsy from 3 o'clock lesion showed fibrocystic changes with sclerosing adenosis and no malignancy cell. Multidisciplinary meeting recommended left wire guided wide local excision with sentinel lymph node sampling with or without axillary clearance depending on the sentinel lymph node findings and postoperative adjuvant chemotherapy and radiotherapy. She underwent wire guided left breast wide local excision with left axillary sentinel lymph node biopsy. The frozen section identified three lymph nodes, one was positive for malignancy, so axillary clearance was done. Final histopathology showed invasive lobular carcinoma, histiocytoid variant, the neoplastic cells exhibited pronounced histiocytoid appearance. The tumor consisted of less than 10% of tumor area forming glandular/tubular structures with cells larger than normal with open vesicular nuclei, visible nucleoli and moderate variability in both size and shape. Immunohistochemical were consistent with the original biopsy findings, tumor cells with abundant granular cytoplasm, Some of the nuclei are irregular, positive Androgen receptor and GCDFP-15 (Fig. 1A-B-C-D). The tumor size was 1 cm and no ductal carcinoma or lobular carcinoma in situ component were identified. All the surgical margins are uninvolved by carcinoma, twelve lymphnodes were identified in the axillary specimen, and all were negative for malignancy (pT1mi, pN1a, Mx). The patient was referred for physical therapy after the operation and was discharged from the hospital day one post-operation with one week follow up in the surgical oncology clinic. After discussing treatment options and risks and benefits, the patient declined adjuvant chemotherapy and agreed to receive adjuvant radiation therapy and continued her regular follow up with the medical oncology. She is currently disease-free 12 months after her surgery confirmed clinically and by ultrasound and mammogram.

## 3 Discussion

Histiocytoid variant of lobular breast carcinoma is an uncommon entity with sparse cases in the literature. After reviewing the English literature, In 1973, Hood et al. described a group of breast carcinomas, showing a predilection for metastasis to the eyelid, composed of cells that resembled histiocytes with small “inactive” nuclei and plentiful vacuolated cytoplasm. The neoplastic cells stained with Mayer mucicarmine and were negative for oil purple O. In five of eight cases on their series, the metastatic lesion manifested earlier than the detection of the primary carcinoma. Those lesions posed difficulties within the differential analysis with different benign lesions, including xanthoma, xanthelasma, histiocytoma, and granular cell tumor [3].

A few authors taken into consideration HBC as a version of apocrine carcinoma or an apocrine variant of infiltrating lobular breast carcinoma based on (1) the consistent expression of the apocrine marker GCDFP-15; (2) the presence of foci of LCIS; (3) the presence of transitional regions between ordinary LCIS and histiocytoid/apocrine LCIS; and (4) the identity of regions with single-mobile filling and targetoid growth pattern in bona fide instances of HBCs [4]. These features and the presence of HBC with pleomorphic lobular carcinoma may suggest that HBC is a version of pleomorphic lobular carcinoma [5]. Later, Gupta et al. described a series of 11 HBCs, eight cases were related to LCIS. In that series, 10 of the eleven reports were positive for the apocrine marker GCDFP-15, and 8 cases lacked E-cadherin expression [6]. Based on these findings, the authors concluded that HBCs have an immunophenotypical profile consistent with each ductal and lobular differentiation and that the shortage of specific and constant medical findings, morphologic appearances, or immunohistochemical profile warrants that histiocytoid carcinoma should not be reported as a special type of breast cancer. In 1989, two cases of in situ and invasive histiocytoid breast carcinoma were also described by N. walford et al. They concluded that histiocytoid carcinoma is considered an apocrine variant of lobular carcinoma and differentiating it from chronic sclerosing inflammation can be challenging in both primary and secondary lesions [4]. In 1995, Eusebi et al. reported thirteen of breast carcinomas all of which have neoplastic cells with granular to foamy cytoplasm. Immunocytochemical and in situ hybridization studies showed that these cells exhibit apocrine differentiation. He concluded that these tumors were easily misinterpreted as either fibrohistiocytic or myoblastomatoid (granular cell) tumors. Therefore, the description of histiocytoid invasive carcinomas was the most appropriate for this specific group of apocrine carcinomas [7]. Similarly, Murali et al. described HBC as breast carcinoma composed mostly or exclusively of cells with foamy and/or granular cytoplasm [1]. A later report by Yu et al. defined the histiocytoid morphological characteristics as having more than 10% tumor cells with small nuclei, inconspicuous nucleoli and granular to foamy cytoplasm with vacuoles or occasional intracytoplasmic vacuoles [8]. The apocrine nature of HBC was based on immunohistochemical positivity for GCDFP-15. Dixon et al. proposed the concept of a pleomorphic variant of invasive lobular carcinoma [9]. However, Kasashima et al. suggested it was a unique entity due to its poor prognosis and mucin expression compared to classical invasive lobular cancer [10].

The recognition of histiocytoid breast carcinoma is often a challenge, particularly when histiocytoid tumor cells occur in a metastatic site before the primary diagnosis of breast cancer, or in insufficient breast biopsy [2.11]. In the breast, uncharacterized histological appearances of the HBC can result in a misdiagnosis with other benign lesions [5]. Its resemblance to benign lesions is a risk, and awareness of these mimics is required in order to safely make a correct diagnosis and then appropriate patient management [11].

An awareness of histological features are needed to make the accurate diagnosis of this condition that should be appropriately subsumed within the invasive lobular histological subtype [12]. Hints to make a correct diagnosis include the presence of accompanying tumor cells that are more pleomorphic and mitotically active, cells with cytoplasmic vacuoles and targetoid secretions, architectural patterns resembling those of invasive lobular cancer with linear files and concentric encirclement of lobules, associated classic invasive lobular carcinoma or lobular neoplasia, and the use of adjunctive immunohistochemistry to verify the epithelial origin of lesioned cells. Clinico-radiological correlation is critical, as discordant findings on core biopsy or cytology should prompt histological pursuit of a conclusive diagnosis on open excision [11].

Metastases to skin can be missed as benign sinus histiocytes and xanthomatous dermal lesions. In the presence of earlier breast cancer, the development of skin nodules comprising histiocyte-like cells should also be diligently assessed to rule out metastasis [11].

## 4 Conclusion

HBC is an unusual tumor that is often regarded as a variant of invasive lobular cancer. The challenging histological appearances may look like benign breast conditions, which poses diagnostic pitfalls, especially on limited material such as core biopsies or fine needle aspiration cytology. Clues to the correct diagnosis are the presence of cells with more overt cytological atypia and mitosis, cells with cytoplasmic vacuoles and secretions, accompanying components of classic invasive lobular carcinoma and/or lobular neoplasia, aided by immunohistochemistry to confirm the epithelial nature of the histiocytoid cells. Close clinic-radiological correlation is essential, as any discordance should trigger further diagnostic determination.

## Funding

This study did not receive any funding.

## Ethical approval

IRB approval is not needed for Case reports in our center.

## Author contribution

Fozan A. Aldulaijan_,_ Abdullah G. Alsahwan, Maryam Hussain A. Alsulaiman_,_ Miral Mohamed Mashhour_,_ Ahmad Alwabari: Study concept and design, data collection, data interpretation, literature review, drafting of the paper, final review of the manuscript.

## Guarantor

Dr. Fozan A. Aldulaijan.

## Declaration of competing interest

No declarations of interest.
